# Caregiver-Level Mental Health as a Contextual Effect in the Association Between Generalized Anxiety Disorder and Suicidal Ideation Among Adolescents: A Multilevel Analysis

**DOI:** 10.1155/2024/5395654

**Published:** 2024-11-25

**Authors:** Zhaohe Zhou, Yi Xu, Dan Luo, Hao Hou, Mengqin Ao, Shuo Liu, Qian Liu, Huijing Zhou, Xiaoqin Wang, Bing Xiang Yang, Yang Zhou, Jun Zhao

**Affiliations:** ^1^School of Basic Medical Sciences/School of Nursing, Chengdu University, Chengdu 610106, Sichuan Province, China; ^2^Center for Wise Information Technology of Mental Health Nursing Research, School of Nursing, Wuhan University, Wuhan, Hubei Province, China; ^3^Office of Psychosocial Services, Wuhan Mental Health Center, Wuhan 430012, Hubei Province, China; ^4^Office of Psychosocial Services, Wuhan Hospital for Psychotherapy, Wuhan 430012, Hubei Province, China

**Keywords:** adolescents, anxiety, caregiver, mental health, multilevel analysis, suicidal ideation

## Abstract

**Background:** Suicidal ideation poses a significant risk for suicidal behavior among adolescents globally. While studies have highlighted the association between anxiety and suicidal ideation, little is known regarding these behaviors in the Chinese adolescent population, as well as possible contextual factors that may influence this relationship. This study explores the association between generalized anxiety disorder (GAD) and suicidal ideation in an urban Chinese in-school adolescent population and investigates the contextual influence of primary caregiver mental health, considering comorbid symptoms of depression, anxiety, and insomnia.

**Methods:** Data from the Students' Mental Health Network (SMHN) project in Wuhan, China, involving 7967 adolescents and their primary caregivers, were analyzed. The study assessed adolescents' and primary caregivers' mental health, including depression, insomnia, and GAD. Suicidal ideation was measured using the 9th item of Patient Health Questionnaire-9 (PHQ-9). Multilevel ordinal logistic regression models were used to examine associations between GAD and suicidal ideation, adjusting for covariates. Contextual effects were explored to determine how the mental health of the primary caregiver influences suicidal ideation.

**Results:** The study found that GAD is independently associated with suicidal ideation among adolescents after accounting for other mental health covariates. Specifically, adolescents with more severe GAD symptoms, particularly subtype symptoms of “Restlessness” and “Irritability,” had a higher likelihood of experiencing suicidal ideation. There were significant contextual effects on adolescent suicidal ideation at the caregiver mental health level. Adolescents with caregivers experiencing depression and anxiety symptoms had an increased risk of suicidal ideation.

**Conclusions:** This research highlights the importance of recognizing GAD as an independent risk factor for suicidal ideation among Chinese adolescents. It also emphasizes the role of caregiver mental health as a contextual factor. The findings suggest a need for holistic interventions addressing both adolescents' and caregivers' mental well-being, ultimately enhancing suicide prevention efforts.

## 1. Introduction

Suicide is the second leading cause of death among adolescents globally [1]. Suicidal ideation, defined as contemplating self-harming behavior, significantly increases the risk of subsequent suicide among adolescents. A meta-analysis of 71 studies has demonstrated a strong association between suicidal ideation and suicide, with an odds ratio (OR) of 3.41 and a 95% confidence interval (CI) ranging from 2.59 to 4.48 [2]. In China, the prevalence of suicidal ideation ranges from 10.7% to 24.2%, with no significant difference between urban and rural areas [3]. Rates of suicidal ideation increase from early adolescence through young adulthood, peaking at the age of 15 [4]. Studies have shown that the presence of suicidal ideation in adolescence may lead to actual suicidal behavior in latter adulthood [5, 6]. Therefore, examining the risk factors associated with suicidal ideation, as well as contextual factors that may account for the inherent variability in suicidal ideation among Chinese adolescents, is of importance to healthcare providers.

One area receiving increasing attention is the link between anxiety and suicidal ideation among adolescents. The role of anxiety in suicidal ideation has been normally examined in conjunction with other mood disorders among adolescents [7–10], but few studies have established a direct link [11], and even fewer have focused on the Chinese population.

Given evidence suggesting that anxiety is a potential risk factor for suicidal ideation, whether acting alone or in combination with other mood disorders, it is important to explore contextual factors that influence this relationship. One theory that may guide research on contextual factors is the Interpersonal-Psychological Theory of Suicide (IPTS) [12]. According to the IPTS, constant and stable thwarted belongingness and perceived burdensomeness are proximal and sufficient causes of suicidal ideation. Thwarted belongingness includes experiences of loneliness and the absence of reciprocal care, such as a nonintact family, social withdrawal, and inadequate parenting practices [13]. Perceived burdensomeness primarily refers to feelings of liability and self-hate [13], which leads individuals to mistakenly interpret their self-hatred as feelings of expendability. As suggested by this theory, the absence of reciprocal care and social burdensomeness could influence suicidal ideation. For adolescents, receiving reciprocal care and a sense of belongingness from primary caregivers are particularly important. However, given the social challenges commonly associated with anxiety disorder, these conditions may be difficult for adolescents to attain. These social challenges include difficulties in social interactions and family relationships, such as social withdrawal due to fear of judgment, communication difficulties that lead to misunderstandings, and low self-esteem that makes individuals feel unworthy of care. Additionally, the fear of negative evaluation and hypervigilance in social situations can make interactions stressful and exhausting, while difficulty in trusting primary caregivers due to constant worry can hinder the formation of supportive relationships. Therefore, research needs to consider contextual effects within the context of caregivers' influence on adolescents, taking into account the possible strain that adolescents' suicidal ideation may stem from their caregivers' mental status. Consistent with the IPTS, the purpose of this study was to examine whether the mental health status of primary caregivers, including depression, anxiety, insomnia, and their comorbidities, as the significant contextual effect that captures the variation in suicidal ideation among adolescents in a school setting in Wuhan, a city in central China.

The mental health status of primary caregivers has both a direct and indirect relationship with adolescents' anxiety symptoms and suicidal ideation. Research has shown that caregivers with mental health problems may not exhibit quality parenting practices [14]. One systematic review based on 19 longitudinal studies concluded that negative parenting practices possibly have an indirect impact on adolescent anxiety [15]. On the other hand, Kawabe et al. [16] established a direct association between caregiver mental health and suicidal ideation among adolescents in Japan.

Based on current evidence, it appears that adolescent anxiety and suicidal ideation are interconnected, and the mental status of the primary caregiver is related to both anxiety and suicidal ideation. Thus, the mental status of the primary caregiver may increase or decrease the odds of suicidal ideation among anxious adolescents, other than the risk factors from the adolescents themselves. The goal of this study is to explore whether the mental status of the primary caregiver acts as a contextual effect in the association between anxiety (and its subtype features) and suicidal ideation among in-school-age adolescents. In this study, the hypothesis is that a primary caregiver with mental health symptoms would serve as a contextual risk factor, elevating the final suicidal ideation risk of an adolescent, while primary caregivers without any mental symptoms would serve as a contextual protective factor. Finally, considering the common co-occurring symptoms of depression that have been adjusted for in previous research [7–11], both the severity of depression and insomnia in adolescents were controlled to provide a conservative test of the study hypothesis.

## 2. Methods

### 2.1. Study Participants

Data were obtained from the Students' Mental Health Network (SMHN), a project involving students in junior and senior high schools in Wuhan. The aim was to explore risk factors associated with adolescent suicidal tendencies and provide evidence for interventions by healthcare professions related to suicide prevention in this age group. The study protocol (No: KY2021.11.01) received approval from the Wuhan Municipal Health Commission and was conducted in accordance with the Declaration of Helsinki.

During the baseline survey, researchers paired students from eight pilot schools with one of their primary caregivers between September and November 2021. Participants were asked to provide online informed consent before dedicating 12–15 min to complete online questionnaires using individualized identification codes. All responses were recorded anonymously for the sole purpose of data analysis. The online platform reviewed the completed questionnaires, resulting in a total of 7967 valid questionnaires.

### 2.2. Instruments

A researcher-designed questionnaire was used to obtain sociodemographic characteristics, including junior high school or high school, sex, one-child family (yes or no), family history of mental illness (anyone in your immediate family ever diagnosed with a mental/psychiatric illness? yes or no), left-behind experience (yes or no), family structure (nuclear family: two parents and their child or children; stem family: grandparents, parents, and their child or children; single-parent family: one parent with one or more children; reorganization family: two divorced parents with one or more children; or grandparent family: one or more grandparents are raising their grandchild or grandchildren), family income (less than ¥80,000/year, ¥80,000–¥150,000/year, ¥150,000–¥300,000/year, or more than ¥300,000/year), and parents' education level (less than high school, high school or college, master's degree or above, or not sure).

Adolescent mental health literacy was assessed using the knowledge section of the 2019 National Mental Health Literacy Questionnaire, developed by the Institute of Psychology, Chinese Academy of Sciences [17]. This segment contains 20 judgment-based questions. Participants received five points for correct responses and 0 points for incorrect or unanswered questions. The maximum achievable score was 100 points, with higher scores indicating a greater level of mental health literacy.

Family functioning was evaluated using the Family APGAR scale [18], which comprises five items, each rated on a 3-point Likert-type scale ranging from “hardly ever” to “almost always.” The five parameters assessed include adaptability, partnership, growth, affection, and resolve. Scores on this scale can range from 0 to 10, reflecting the level of satisfaction with family functioning and emotional support within family life, with higher scores indicating greater satisfaction. The reliability and validity of this instrument have been well-established by Smilkstein, Ashworth, and Montano [19]. The Cronbach *α* coefficient for this scale in the present study was 0.889.

Adolescent depression was assessed using the Patient Health Questionnaire-2 (PHQ-2) [20], a self-assessment tool comprising two items that gauge the severity of depression symptoms in the past 2 weeks. Respondents rate each item on a 4-point Likert scale, ranging from “not at all” to “nearly every day.” The total score on this scale can range from 0 to 6, with higher scores indicating greater severity of depressive symptoms. Separately, primary caregiver depression was assessed using the Patient Health Questionnaire-9 (PHQ-9) [21], a self-assessment tool comprised of nine items that assesses the severity of anxiety symptoms in the past 2 weeks. The total score on this scale can range from 0 to 27. The Chinese version of the PHQ-9 has demonstrated robust reliability and validity in previous studies [22]. The Cronbach *α* coefficients for these scales in the present study were 0.802 and 0.874 for adolescents and primary caregivers, respectively.

Adolescent and primary caregiver insomnia were assessed using the Insomnia Severity Index-7 (ISI-7) [23], a self-assessment tool comprised of seven items that gauge the severity of insomnia symptoms in the past 2 weeks. The responses are rated on a 4-point Likert scale from “none” to “very severe.” The total score on this scale ranges from 0 to 28, with higher scores indicating greater severity of insomnia symptoms. The Chinese version of the ISI-7 has been confirmed to possess both validity and efficacy [24]. The Cronbach *α* coefficients for these scales in the present study were 0.859 and 0.878 for adolescents and primary caregivers, respectively.

Adolescent and primary caregiver anxiety were assessed using the General Anxiety Disorder-7 (GAD-7) [25], a self-assessment tool comprised of seven items that gauge the severity of GAD symptoms in the past 2 weeks. Respondents rate each item on a 4-point Likert scale, ranging from “not at all” to “nearly every day.” The total score on this scale can range from 0 to 21, with higher scores indicating greater severity of GAD symptoms. The Chinese version of the GAD-7 has also demonstrated good psychometrics and can serve as a tool for anxiety screening [26]. To further explore the subtype features of GAD, seven key symptoms were derived from the seven questions of the GAD-7 measurement tool to access the frequency and severity for each specific dimension of GAD. The subtype features are “Nervousness” (GAD1), “Uncontrollable worry” (GAD2), “Difficulty shifting attention” (GAD3), “Trouble relaxing” (GAD4), “Restlessness” (GAD5), “Irritability” (GAD6), “Excessive fear” (GAD7), with higher scores indicating greater severity of that subtype. The Cronbach *α* coefficient of these scales in the present study were 0.921 and 0.915 for adolescents and primary caregivers, respectively.

Primary caregiver-level mental health combined the presence of three possible symptoms: depression, insomnia, and anxiety, where the effect of each symptom was determined by the recommended cutoff points of the measurements described above [21, 23, 25]. This identifies eight possible types of caregiver-level mental status: no mental symptoms, depression, anxiety, insomnia, comorbidity of depression and anxiety, comorbidity of depression and insomnia, comorbidity of anxiety and insomnia, and comorbidity of depression, anxiety, and insomnia. In this study, the researchers considered primary caregiver-level mental status as a contextual phenomenon, given the clustering of individual adolescents living within the same caregiving environment.

Coronavirus disease 2019 (COVID-19)-related lockdown measures were not included in the study for several reasons. First, the data collection for the program occurred in September and October 2021, after the Chinese government lifted the lockdown in Wuhan. Following the lockdown, Wuhan residents were permitted to travel outside the city and return to work. Zhou et al. [27] conducted a four-wave questionnaire study to examine how Wuhan residents' psychological experiences evolved within the first 2 months after the lockdown was lifted. The study generally found that the public perceived a gradual return to normalcy, with the structure of everyday life being restored and psychological well-being improving after the lockdown ended. Additionally, a cross-sectional online study assessing Wuhan residents' mental health post-lockdown indicated similar findings: the proportion of anxiety and depression among the general public decreased compared to the lockdown period [28]. Although COVID-19 remained a global pandemic, daily life in Wuhan had largely returned to normal by the time of the data collection period. Second, while the COVID-19 pandemic created significant uncertainty worldwide, this impact was not solely due to lockdown measures. The effects were felt on a broader social level, influencing everyone. In this study, the focus was on family dynamics, which is why COVID-19-related lockdown measures were not considered.

### 2.3. Measurement of Suicidal Ideation

The primary outcome of this study was to assess adolescent suicidal ideation over the past 2 weeks, measured using the final item of the PHQ-9. The PHQ9 is a widely recognized tool in research for identifying the presence of suicidal ideation [29, 30], known for its robustness as an indicator of suicide risk [31]. This item specifically evaluates the frequency of thoughts related to death and self-harm in the preceding 2 weeks, making it a commonly used scale in studies examining the prevalence of suicidal ideation [27, 28, 32]. Respondents provided their ratings on a 4-point Likert scale, ranging from “not at all” to “nearly every day.” Following the recommendation of the American Heart Association Science Advisory, which suggests that all participants who respond “Yes” on the PHQ9 should be promptly assessed for suicide [33]. Therefore, a response of “not at all” was categorized as indicating no suicidal ideation. A response of “several days” was considered as indicating “Yes” and was classified as less frequent suicidal ideation. Responses of “More than half the days” and “nearly every day” were classified as indicating more frequent suicidal ideation.

### 2.4. Statistical Analysis

For descriptive statistics, frequencies (proportions) for categorical variables or mean values (standard deviation) for continuous variables were used to describe participant characteristics and suicidal ideation.

To investigate the associations between GAD and suicidal ideation, considering the contextual effect of caregiver-level mental health, multilevel ordinal logistic regression models were used to estimate the adjusted ORs (aORs), along with their corresponding 95% CIs and *p* values. Regarding GAD in adolescents exhibiting suicidal ideation, four models were constructed. The null model (Model 0), devoid of any variables, was formulated to decompose the amount of variance that existed in the mental status of the caregiver. The first model (Model 1) assessed the fixed effects of sociodemographic variables, family function, and adolescent mental health literacy on suicidal ideation, presented as aORs with their 95% CIs. The second fully adjusted model (Model 2) additionally assessed the fixed effects of all mental covariates among adolescents, encompassing depression severity, insomnia severity, and GAD severity. The third model (Model 3), instead of adjusting GAD severity, broke down the fixed effects of all seven subtype features of GAD symptoms in relation to suicidal ideation. Before the regression analysis, the Pearson correlation matrix and the variance inflation factors (VIFs) based on Model 2 were calculated to examine the presence of multicollinearity between explanatory variables. To control the risk of type 1 error, the Ryan–Holm step-down Bonferroni procedure was used to adjust all *p* values [34] as well as the 95% CIs of the estimates [35].

To elucidate the contextual impact of caregiver-level mental health on suicidal ideation, the random intercepts and their respective 95% CIs for the fully adjusted model were estimated (Model 2). If a 95% CI of random intercept does not include zero, it provides statistical support (at the 5% significance level) that adolescent suicidal ideation risk from one specific type of caregiver-level mental status was different from overall average suicidal ideation risk. Model evaluation metrics included variance, proportional change in variance, intraclass correlation coefficient (ICC) [36], and median OR (MOR), which are widely used for assessing clustering effects and unexplained cluster heterogeneity [37, 38].

All computations were performed using R (version 3.5.0); the R package “ordinal” was employed for performing multilevel ordinal logistic regression models. All tests were two-sided and considered statistically significant if the *p*-value was <0.05.

## 3. Results

### 3.1. Descriptive Statistics

The current study included 7967 adolescents and their primary caregivers, consisting of 3668 female participants (46.0%) and 4299 male participants (54.0%) in the final analysis. More than half (52.1%) of the participants were high school students, 62.4% were the only child in their family, 89.6% had childhood left-behind experience, and 54.9% were from nuclear families.

In total, 1021 participants (12.8%) experienced suicidal ideation. Among them, 741 participants met the criteria for being classified as less frequent suicidal ideators, while 280 participants met the criteria for being classified as more frequent suicidal ideators. This translates to prevalence rates of 9.3% for less frequent suicidal ideation and 3.5% for more frequent suicidal ideation among the sample. Notably, female participants exhibited higher prevalence rates for less frequent and more frequent suicidal ideators. Further details can be found in Table 1.

### 3.2. Association Between Anxiety and Suicidal Ideation

After sociodemographic characteristics, socioeconomic status, family environment, and other psychiatric covariates were controlled (Model 2), an increase in the odds of suicidal ideation was positively associated with an increase in the GAD score (aOR = 2.001, 95% CI = 1.730–2.316, *p*  < 0.001). The more severe the GAD, the higher the degree of suicidal ideation in the adolescents sample. Model 3 further examines the association between each of the seven subtype features of GAD and suicidal ideation. Scores for “Restlessness” (aOR = 1.228, 95% CI = 1.015–1.486, *p*=0.022), “Irritability” (aOR = 1.281, 95% CI = 1.059–1.550, *p*=0.001), and “Excessive fear and tension” (aOR = 1.433, 95% CI = 1.221–1.682, *p*  < 0.001) were significantly different between adolescents with more frequent suicidal ideation and less frequent suicidal ideation and those without suicidal ideation. The aORs did not change substantially from Model 1 to the fully adjusted models (Model 2 and Model 3). After adjusting for variables, being a junior high student, sex, family dysfunction, depression severity, and insomnia severity remained significant in Model 2 and Model 3 after applying the Bonferroni procedure. The detailed aORs, 95% CI, and *p* values for all three models are presented in Table 2. The Pearson correlation coefficients and VIFs for all independent variables are presented in Tables S1 and S2.

### 3.3. Variation in Caregiver-Level Mental Status

**Figure 1:**
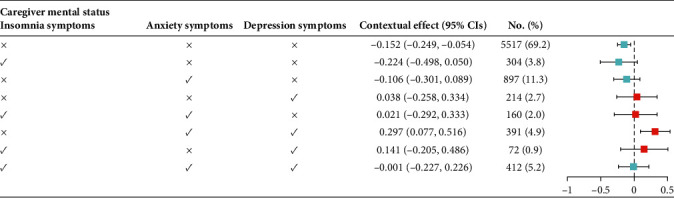
Contextual effects of caregiver-level mental status. CIs, confidence intervals.

Table 3 and Figure 1 show the random effect (measure of variation) results from multilevel analysis. Eight caregiver-level mental comorbidities were tested as a contextual factor to present the potential effect of caregiver-level mental comorbidity applied to individual adolescent suicidality, other than risk factors regularly tested in adolescents. The MORs greater than one for all models provide evidence of the contextual phenomenon that caregiver-level mental status modified the likelihood of suicidal ideation among adolescents. The MOR of 1.44 (null model) between adolescents with a higher and lower propensity of suicidal ideation within a caregiver-level suggested the heterogeneity between different caregiver mental comorbidity is moderate (Table 3). After accounting for individual-level covariates, the fully adjusted models reduced the unexplained heterogeneity among caregiver-level factors, resulting in an MOR of ~1.2. According to the proportional change in variance presented in Table 3, ~65.4%, 67.1%, and 69.5% of the variance in the log odds of adolescent suicidal ideation across caregiver-level were explained by Model 1–Model 3, respectively. The ICC implied by the estimated intercept component variance for caregiver-level mental status showed that 4.2% of the variance of adolescent suicidal ideation could be linked to the caregiver-level mental status in the null model (Model 0) and about 1.4% in the fully adjusted model (Model 2). As for the actual effects, the majority of caregivers who did not have any depression, anxiety, and insomnia symptoms (*n* = 5517, 69.2%) had a random effect on the intercept of −0.152 (95% CI, −0.249—−0.054), which indicates that a caregiver without any mental symptoms tends to attenuate the association between anxiety and suicidal ideation. Caregivers who had a mental comorbidity of depression and anxiety (*n* = 391, 4.9%) had a random effect on the intercept of 0.297 (95% CI, 0.077—0.516), which indicates that a caregiver with depression and anxiety symptoms tends to confer additional risk and strengthen the relationship between anxiety and suicidal ideation. The detailed random effects on the intercept and their 95% prediction interval are presented in Figure 1.

## 4. Discussion

This cross-sectional survey revealed that suicidal ideation was experienced by 12.8% of school adolescents in the preceding weeks, which includes 3.5% classified as frequent suicidal ideators. The study identified that the mental status of adolescents, particularly the symptoms of GAD, along with the mental comorbidity of caregivers, is associated with suicidal ideation among Chinese adolescents. In comparing the mental status of adolescents, the study unveiled the partial roles played by the mental comorbidity of caregivers in relation to suicide outcomes. To the best of the researchers' knowledge, this study represents the first attempt to examine the relationship between suicidal ideation and GAD symptoms in adolescents while incorporating caregiver mental health as a contextual effect in the analysis.

### 4.1. Explanations of Associations

The link between suicidal ideation and anxiety has traditionally been explored within a larger body of context on mood disorders [7–9], particularly major depressive disorder co-occurring with GAD [10]. However, some studies highlighted the independent risk factor of anxiety disorders for suicidal ideation among adults. Sareen et al. [39] were among the first to demonstrate this link in an adult population, while Gilmour [40] further aids the independent association between threshold and subthreshold GAD and suicidal ideation. Boden, Fergusson, and Horwood [11] conducted a longitudinal study that revealed the steady impact of GAD on suicidal ideation from late adolescence to young adulthood, even after adjusting for common psychiatric covariates. These findings underscore the importance of recognizing GAD as an independent risk factor for suicidal ideation. The present study supports these findings in adolescents in a Chinese population. This study also found a moderate independent association between GAD severity and suicidal ideation among adolescents, even after controlling for other psychiatric symptoms such as depression and insomnia. The results suggest that adolescents with more severe GAD symptoms were twice as likely to experience suicidal ideation, which is consistent with the results previously reported by Sareen et al. [39] and Boden, Fergusson, and Horwood [11] (aORs 2.3 and 2.8, respectively). Overall, these findings suggest that early interventions targeting anxiety disorders, particularly GAD, could play a crucial role in reducing suicide risk among adolescents.

The present study revealed a unique association between GAD and suicidal ideation, prompting the researchers to explore the specific symptoms by which GAD and suicidal ideation are associated. Two out of three significant symptoms were found to be the physical sensations of “Restlessness” and “Irritability.” While previous community-based [41, 42] and clinical-based [43] studies have shown promising results linking “Irritability” to suicidal ideation in adolescents; evidence for the association between “Restlessness” and suicidal ideation is scant due to the lack of systematic assessment of the symptoms in adolescents. In school environments, it is recommended that both teachers and caregivers pay attention to individuals who exhibit irritability and related physical sensations when assessing suicide risk, as irritable individuals may have reduced social support [44]; therefore, additional support from schools and families is crucial in addressing this issue. Future research should expand upon the current study by including more comprehensive physical dysregulations, such as anger and impulsive aggression, to enhance understanding of these relationships.

The present study aimed to explore whether GAD affects suicidal ideation among adolescents. To minimize the risk of excessive type 1 error that may occur when many other potential factors were tested in a single model, the current study used the Ryan–Holm step-down Bonferroni procedure to control for false positive results. Besides the link between GAD severity and suicidal ideation, being a junior high student, sex, family dysfunction, depression severity, and insomnia severity came out as significant risk factors with suicidal ideation among adolescents after the Bonferroni correction. The risk factors are highly consistent with those of earlier adolescent-related suicidality literature; several risk factors include being female [9, 45, 46], self-perceived family dysfunction [9, 47–49], depression severity [8, 42, 50], and insomnia severity [51, 52]. The other risk factor of being a junior high school adolescent was not explicitly identified in other similar studies due to differences in the age measurement instruments used. However, several studies do align on the age cutoff, recognizing that the transition from junior high school to senior high school typically occurs around the age of 15 in China [9, 44]. This difference may stem from the fact that senior high school students generally exhibit greater cognitive and emotional maturity compared to junior high school students. This maturity often translates into more effective coping mechanisms for managing psychological distress and reducing the likelihood of suicidal ideation. According to Erikson's theory of psychosocial development [53], high school adolescents are navigating the “Identity vs. Role Confusion” stage, which is pivotal in developing a strong sense of self. Successfully resolving the challenges of this stage can diminish feelings of hopelessness and mitigate the risk of suicidal thoughts. In contrast, junior high school students are in the process of transitioning from the “Industry vs. Inferiority” stage, a period where they are more susceptible to feelings of inadequacy and vulnerability, potentially exacerbating the risk of suicidal ideation.

### 4.2. Explanations of the Variation

Based on the epidemiological concept of contextual influence and the principle of multilevel analysis [36], individuals within the same “cluster” are expected to exhibit greater similarity in terms of health outcomes compared to individuals from different “clusters.” This similarity can be attributed to various factors, such as the mental status of primary caregivers, to which individual adolescents are more susceptible. Owing to this contextual effect, a portion of health outcomes may be linked to the specific cluster in which individuals reside. Building upon this theoretical foundation, the study's findings suggest that variation in caregiver-level mental comorbidity contextual factor influencing adolescent suicidal ideation. Approximately 4% of the total individual variance in suicidal ideation was attributed to differing caregiver-level mental status. Taking these variances into account, adolescents whose primary caregivers experience depression and anxiety have an increased risk of suicidal ideation.

One possible explanation is that adolescents are particularly affected by anxiety and depression disorders [54]. Recent research [55–57] has shown that poor mental health, especially symptoms of anxiety and depression, among primary caregivers are associated with poor mental health among their children, thereby increasing the risk of suicidal ideation [58, 59]. The mental comorbidity of depression and anxiety in primary caregivers may also exert an indirect effect on adolescent suicidal ideation. Based on the research instruments [21, 25] used in the study, anxiety, and depression often manifest as pronounced and persistent signs, such as excessive worry, intense fear, low mood, loss of interest, and irritability. These emotional and behavioral changes, linked to symptoms among caregivers, may negatively impact adolescent suicidal tendencies indirectly by contributing to family dysfunction [43, 60] and family environment [44, 45, 61] in general. Research conducted by Kwok and Shek [62] explored the relationship between family function and suicidal ideation in Hong Kong adolescents, reporting that parent-adolescent communication serves as a significant protective factor against suicidal ideation for both sexes. Family discord and poor parental relationships have also been associated with an increased likelihood of suicidal ideation among adolescents [63, 64]. Garrison et al. [65] further documented that family cohesion and adaptability were consistently linked to heightened suicidal ideation, supported by both cross-sectional and longitudinal models. In conclusion, these variations underscore the distinct moderating effect of caregiver mental comorbidity in relation to suicidal ideation among adolescents.

The findings indicate that comorbid depression, anxiety, and insomnia in the primary caregiver do not significantly moderate the relationship between adolescent anxiety and suicidal ideation. This could be due to the nature of insomnia, which often coexists with psychiatric disorders such as anxiety and depression. Studies show that insomnia co-occurs with these conditions in at least 40%–50% of the general adult population [66, 67]. Population-based cohort study aiming to identify risk factors for depression and anxiety has shown that insomnia is a state marker of current anxiety and depression, meaning it appears when these disorders are present [68]. Earlier clinical practice and diagnostic frameworks, such as the Diagnostic and Statistical Manual of Mental Disorders (DSM) 3rd and 4th editions [69, 70], conceptualized insomnia as a secondary symptom of primary disorders like depression and anxiety. The interaction between insomnia and other mental health conditions remains complex. Insomnia's role as a secondary symptom or as part of a comorbid condition can obscure its unique contribution to mental health outcomes. The lack of a significant moderating effect in this study might be attributed to this complexity, where the overlapping symptoms of insomnia, anxiety, and depression make it difficult to isolate the specific influence of insomnia. In recent decade, researchers have noticed a shift in paradigms with the advent of the revised classification of sleep disorders, most notably in the DSM 5th and the International Classification of Sleep Disorders (ICSD) 3rd [71, 72], which recognizing insomnia as a disorder on its own. This shift has allowed professionals to classify and treat insomnia subtypes independently, providing a more nuanced understanding of its impact. Hence, future research should focus on delineating the subtypes of insomnia and their specific impacts when comorbid with anxiety and depression. By applying the newest diagnostic paradigms, researchers can better understand how different manifestations of insomnia among primary caregivers might moderate the relationship between adolescent anxiety and suicidal ideation. This approach could uncover more precise mechanisms and inform targeted interventions for at-risk adolescents.

The findings in this study emphasize the need for a holistic approach to adolescent mental health care that considers the mental well-being of primary caregivers. Integrating caregiver mental health assessment and support into adolescent mental health programs can lead to more comprehensive and effective interventions. By addressing caregiver mental health, teachers and healthcare professionals can create a supportive environment that promotes the overall well-being of both adolescents and their caregivers. In the school settings, educators and administrators should consider implementing screening programs that identify both adolescents with mental health concerns and their caregivers experiencing depression and anxiety. By identifying these risk factors early, school personnel can facilitate timely interventions and connect students and families with appropriate mental health resources.

### 4.3. Limitations and Strengths

There are several limitations of this study. First of all, The sample consisted of students and their primary caregivers from junior and senior high schools in Wuhan. As a result, caution must be exercised when interpreting the results, given that the study's focus centers on an urban adolescent population. Nonetheless, a substantial sample of adolescents, paired with their primary caregivers across eight junior and senior high schools, was used, which reflects the social, economic, and cultural norms characteristic of a typical large city in China. This study focused on understanding the role of individual and primary caregiver mental status as correlates of suicidal ideation. No assessment of the interaction effect between variables was incorporated. Apart from individual and caregiver factors, external influences such as the school environment, including teacher–student relationships, peer interactions, academic stress, etc., could also impact individuals' suicidal thoughts. The survey used in this study reported the mental status of adolescents and their primary caregivers over a 2-week duration. Consequently, evaluating mood changes over an extended period was not feasible. Longitudinal studies can provide valuable insights into the trajectories of both adolescent and caregiver mental health and help tailor interventions to evolving needs.

Despite these limitations, the study also has significant strengths. Among the first of its kind, this study incorporates the mental status and comorbidity of three factors related to adolescent primary caregivers into the analysis. Utilizing a multilevel modeling approach to study contextual effects that influence adolescent suicidal ideation has its advantages. In particular, the mental comorbidity of primary caregivers aids in inferring the caregiving environment in which individuals reside and experience health outcomes. Another strength lies in the extensive adjustment of different potential confounding variables related to the primary study variable in the model. However, the researchers cannot entirely dismiss the potential for residual confounding due to unknown factors or measurement errors introduced by the instruments employed in the study. Given the observed independent association between anxiety and suicidal ideation in the sample, schools should exercise particular caution when assessing suicide risk in adolescents with anxiety disorders. Furthermore, Gould et al. [73] conducted a school-based randomized controlled trial to examine whether asking about suicidal ideation in a screening program increases suicidal ideation among high school students in general or specifically among high-risk students reporting depressive symptoms. The study results demonstrate the absence of an iatrogenic effect of asking about suicide among in-school adolescents. Hence, this study minimizes potential information biases.

## 5. Conclusions

The study provides insights into the independent relationship between GAD and suicidal ideation among Chinese adolescents. It introduces a unique perspective by incorporating caregiver-level mental health as a contextual factor, shedding light on its impact on adolescent suicide risk. The results highlight that more severe GAD symptoms, particularly “Restlessness” and “Irritability,” are associated with increased suicidal ideation. Caregiver mental status, specifically the comorbidity of depression and anxiety, is an important correlate of adolescent suicidal ideation in China. Findings emphasize the necessity for comprehensive interventions that encompass both adolescent and caregiver mental well-being, enhancing the effectiveness of preventive strategies. Policymakers and educators should collaborate to implement early identification and support programs, ultimately fostering a healthier environment for both adolescents and their primary caregivers.

## Figures and Tables

**Table 1:** Sociodemographic characteristics of adolescents and primary caregivers (*N* = 7967).

Categorical variables	No. (%)	Less frequent (*N* = 741)	More frequent (*N* = 280)
Junior high school			
Junior high school	3814 (47.87%)	327 (44.13%)	163 (58.21%)
High school	4153 (52.13%)	414 (55.87%)	117 (41.79%)
Gender			
Male	4299 (53.96%)	323 (43.59%)	108 (38.57%)
Female	3668 (46.04%)	418 (56.41%)	172 (61.43%)
Paternal education			
High school diploma or below	3795 (47.63%)	335 (45.21%)	117 (41.79%)
Associate or bachelor's degree	3678 (46.17%)	352 (47.50%)	132 (47.14%)
Master's degree or above	488 (6.13%)	52 (7.02%)	31 (11.07%)
Not sure	6 (0.08%)	2 (0.27%)	0 (0.00%)
Maternal education			
High school diploma or below	4165 (52.28%)	366 (49.39%)	123 (43.93%)
Associate or bachelor's degree	3475 (43.62%)	345 (46.56%)	136 (48.57%)
Master's degree or above	322 (4.04%)	29 (3.91%)	21 (7.50%)
Not sure	5 (0.06%)	1 (0.13%)	0 (0.00%)
One-child family			
No	2998 (37.63%)	284 (38.33%)	118 (42.14%)
Yes	4969 (62.37%)	457 (61.67%)	162 (57.86%)
Left-behind experience			
No	824 (10.34%)	106 (14.30%)	54 (19.29%)
Yes	7143 (89.66%)	635 (85.70%)	226 (80.71%)
Family structure			
Nuclear family	4375 (54.91%)	377 (50.88%)	134 (47.86%)
Stem family	1533 (19.24%)	125 (16.87%)	42 (15.00%)
Single-parent family	1803 (22.63%)	199 (26.86%)	90 (32.14%)
Reorganized family	93 (1.17%)	8 (1.08%)	8 (2.86%)
Grandparent family	163 (2.05%)	32 (4.32%)	6 (2.14%)
Family income			
<¥80,000/year	1804 (22.64%)	153 (20.65%)	62 (22.14%)
¥80,000–¥150,000/year	2936 (36.85%)	278 (37.52%)	92 (32.86%)
¥150,000–¥300,000/year	2242 (28.14%)	194 (26.18%)	82 (29.29%)
>¥300,000/year	985 (12.36%)	116 (15.65%)	44 (15.71%)
Family history of mental illness			
Yes	7589 (95.26%)	678 (91.50%)	248 (88.57%)
No	378 (4.74%)	63 (8.50%)	32 (11.43%)

**Continuous variable**	**Mean ± SD**		

Family function	7.07 ± 2.76	4.89 ± 2.77	3.77 ± 3.05
Adolescent mental health literacy	15.12 ± 2.43	15.70 ± 2.20	14.88 ± 2.51
Adolescent depression (PHQ-2)	1.21 ± 1.39	2.48 ± 1.29	3.92 ± 1.68
Adolescent insomnia (ISI-7)	3.74 ± 4.28	6.99 ± 5.16	10.18 ± 6.32
Adolescent anxiety (GAD-7)	3.80 ± 4.39	8.27 ± 4.65	12.66 ± 5.43
Subtype features of anxiety			
GAD1: Nervousness	0.68 ± 0.74	1.27 ± 0.75	1.96 ± 0.96
GAD2: Uncontrollable worry	0.51 ± 0.75	1.16 ± 0.87	1.86 ± 1.02
GAD3: Difficulty shifting attention	0.60 ± 0.78	1.26 ± 0.88	1.89 ± 0.99
GAD4: Trouble relaxing	0.62 ± 0.83	1.27 ± 0.89	1.87 ± 1.02
GAD5: Restlessness	0.35 ± 0.64	0.88 ± 0.80	1.34 ± 1.03
GAD6: Irritability	0.59 ± 0.79	1.29 ± 0.88	1.96 ± 1.03
GAD7: Excessive fear	0.47 ± 0.78	1.14 ± 0.95	1.78 ± 1.11
Primary caregiver depression (PHQ-9)	0.23 ± 0.42	0.33 ± 0.47	0.35 ± 0.48
Primary caregiver insomnia (ISI-7)	0.14 ± 0.34	0.23 ± 0.42	0.29 ± 0.45
Primary caregiver anxiety (GAD-7)	0.12 ± 0.32	0.15 ± 0.36	0.20 ± 0.40

Abbreviations: %, proportion (percentage); GAD-7, general anxiety disorder-7; ISI-7, insomnia severity index-7; *N*, number of adolescents; PHQ-2, Patient Health Questionnaire-2; SD, standard deviation.

**Table 2:** Factors associated with suicidal ideation identified by multilevel ordinal logistic regression models.

Variable	Model 1^A^		Model 2^B^		Model 3^C^	
Fixed effects	aORs (95% CIs*⁣*^*∗*^)	*p* ^*∗*^	aORs (95% CIs*⁣*^*∗*^)	*p* ^*∗*^	aORs (95% CIs*⁣*^*∗*^)	*p* ^*∗*^
High school (vs. junior high school)	0.760 (0.590–0.978)	0.020	0.736 (0.568–0.954)	0.007	0.745 (0.571–0.972)	0.016
Female (vs. male)	1.523 (1.198–1.935)	<0.001	1.320 (1.034–1.685)	0.012	1.335 (1.035–1.723)	0.012
Paternal education						
High school diploma or below	1 (reference)	—	1 (reference)	—	1 (reference)	—
Associate or bachelor's degree	0.950 (0.712–1.266)	0.998	0.913 (0.669–1.245)	0.992	0.927 (0.682–1.261)	0.996
Master's degree or above	1.040 (0.678–1.595)	0.974	0.980 (0.634–1.514)	0.993	1.021 (0.658–1.582)	0.993
Not sure	10.884 (0.113–1050.477)	0.823	20.017 (0.187–2140.939)	0.576	19.614 (0.163–2359.016)	0.666
Maternal education						
High school diploma or below	1 (reference)	—	1 (reference)	—	1 (reference)	—
Associate or bachelor's degree	1.295 (0.937–1.791)	0.252	1.269 (0.911–1.766)	0.402	1.270 (0.905–1.782)	0.461
Master's degree or above	1.093 (0.605–1.974)	0.997	1.006 (0.634–1.597)	0.981	0.990 (0.621–1.579)	0.967
Not sure	0.012 (0.000–6.686)	0.451	0.009 (0.000–4.618)	0.330	0.008 (0.000–4.879)	0.363
One-child family (vs. no)	1.068 (0.851–1.340)	0.981	1.052 (0.834–1.327)	0.997	1.062 (0.835–1.350)	0.998
Left-behind experience (vs. no)	1.185 (0.845–1.662)	0.843	1.153 (0.815–1.631)	0.953	1.134 (0.795–1.618)	0.991
Family structure						
Nuclear family	1 (reference)	—	1 (reference)	—	1 (reference)	—
Stem family	0.890 (0.654–1.210)	0.956	0.862 (0.623–1.192)	0.919	0.845 (0.605–1.179)	0.894
Single-parent family	1.092 (0.842–1.415)	0.970	1.087 (0.829–1.426)	0.993	1.075 (0.818–1.412)	0.998
Reorganized family	1.030 (0.550–1.931)	0.926	0.923 (0.401–2.121)	0.999	0.924 (0.400–2.134)	0.999
Grandparent family	1.758 (0.888–3.483)	0.211	1.799 (0.900–3.597)	0.178	1.773 (0.874–3.600)	0.250
Family income						
¥80,000–¥150,000/year	1 (reference)	—	1 (reference)	—	1 (reference)	—
Less than ¥80,000/year	0.973 (0.749–1.262)	0.992	1.036 (0.777–1.380)	0.999	1.047 (0.780–1.405)	0.999
¥150,000–¥300,000/year	0.973 (0.753–1.259)	0.998	1.020 (0.795–1.310)	0.996	1.021 (0.794–1.313)	0.996
More than ¥300,000/year	1.153 (0.798–1.665)	0.962	1.075 (0.754–1.534)	0.995	1.054 (0.743–1.495)	0.998
Family function	0.599 (0.528–0.680)	<0.001	0.627 (0.551–0.713)	<0.001	0.629 (0.550–0.719)	<0.001
Family history of mental illness (vs. no)	1.552 (1.001–2.406)	0.048	1.501 (0.958–2.351)	0.115	1.489 (0.941–2.358)	0.162
Adolescent mental health literacy	1.082 (0.955–1.226)	0.603	1.029 (0.911–1.161)	0.997	1.036 (0.913–1.176)	0.998
Adolescent depression (PHQ-2)	2.589 (2.280–2.939)	<0.001	1.886 (1.632–2.179)	<0.001	1.920 (1.650–2.234)	<0.001
Adolescent insomnia (ISI-7)	1.481 (1.328–1.650)	<0.001	1.203 (1.069–1.355)	<0.001	1.204 (1.066–1.361)	<0.001
Adolescent anxiety (GAD-7)	—	—	2.001 (1.730–2.316)	<0.001	—	—
Subtype features of anxiety						
GAD1: Nervousness	—	—	—	—	1.045 (0.845–1.293)	0.998
GAD2: Uncontrollable worry	—	—	—	—	1.234 (0.990–1.539)	0.074
GAD3: Difficulty shifting attention	—	—	—	—	1.096 (0.890–1.349)	0.954
GAD4: Trouble relaxing	—	—	—	—	0.946 (0.794–1.127)	0.996
GAD5: Restlessness	—	—	—	—	1.228 (1.015–1.486)	0.022
GAD6: Irritability	—	—	—	—	1.281 (1.059–1.550)	0.001
GAD7: Excessive fear	—	—	—	—	1.433 (1.221–1.682)	<0.001

Abbreviations: 95% CIs, 95% confidence intervals; aORs, adjusted odds ratios; GAD-7, general anxiety disorder-7; ISI-7, insomnia severity index-7; PHQ-2, Patient Health Questionnaire-2.

Model 1^A^ adjusted for all demographic variables, family function, and adolescent mental health literacy.

Model 2^B^ adjusted for mental covariates of adolescents.

Model 3^C^ adjusted for seven subtype features of GAD symptoms instead of adolescent GAD severity.

*p* ^*∗*^, 95% CIs*⁣*^*∗*^, adjusted for inferences using the Ryan–Holm step-down Bonferroni method.

**Table 3:** Measures of caregiver-level variation for multilevel ordinal logistic regression models.

Measures of variation	Model 0^0^	Model 1^A^	Model 2^B^	Model 3^C^
Caregiver-level				
Variance	0.145	0.050	0.048	0.044
Explained variation (%)	Reference	65.4	67.1	69.5
Intraclass correlation coefficient (%)	4.2	1.5	1.4	1.3
Median odds ratio	1.44	1.24	1.23	1.22

Model 0^0^ is empty model, a baseline model without any variable.

Model 1^A^ is adjusted for sociodemographic variables, family function, and adolescent mental health literacy.

Model 2^B^ adjusted for mental covariates of adolescents.

Model 3^C^ adjusted for seven subtype features of GAD symptoms instead of adolescent GAD severity.

## Data Availability

Raw data were generated at Wuhan Mental Health Center. Derived data supporting the findings of this study are available from the corresponding author Yang Zhou upon request.

## Supporting Information

Supporting Information The file provides additional details supporting the main findings in this study. Table S1. Pearson correlation coefficients for all independent variables included in Model 2, allowing for examination of inter-variable correlations. Table S2. Variance inflation factors (VIFs) for the independent variables in Model 2, which help to assess multicollinearity. These data enhance understanding of the relationships and ensure robustness in interpreting the associations reported in the primary analysis.
