# Supplementary material for: Caregiver-Level Mental Health as a Contextual Effect in the Association Between Generalized Anxiety Disorder and Suicidal Ideation Among Adolescents: A Multilevel Analysis
Source: Depress Anxiety. 2024 Nov 25;2024:5395654. doi: 10.1155/2024/5395654 (PMC11918931; doi:10.1155/2024/5395654)
Supplement: Supporting Information — The file provides additional details supporting the main findings in this study. Table S1. Pearson correlation coefficients for all independent variables included in Model 2, allowing for examination of inter-variable correlations. Table S2. Variance inflation factors (VIFs) for the independent variables in Model 2, which help to assess multicollinearity. These data enhance understanding of the relationships and ensure robustness in interpreting the associations reported in the primary analysis. [file 5395654.f1.doc]

**Supplementary Table 1. Pearson correlation coefficients for all independent variables in model 2**

|  | **High school** | **Gender** | **Paternal education** | **Maternal education** | **One-child family** | **Left-behind experience** | **Family structure** | **Family income** | **Family function** | **Family history of mental illness** | **Adolescent mental health literacy** | **Adolescent depression (PHQ-2)** | **Adolescent insomnia (ISI-7)** | **Adolescent anxiety (GAD-7)** |
| --- | --- | --- | --- | --- | --- | --- | --- | --- | --- | --- | --- | --- | --- | --- |
| **High school** | 1 |  |  |  |  |  |  |  |  |  |  |  |  |  |
| **Gender** | -0.0202 | 1 |  |  |  |  |  |  |  |  |  |  |  |  |
| **P-value** | 0.0716 |  |  |  |  |  |  |  |  |  |  |  |  |  |
| **Paternal education** | -0.0758 | 0.0286 | 1 |  |  |  |  |  |  |  |  |  |  |  |
| **P-value** | < 0.001 | 0.0107 |  |  |  |  |  |  |  |  |  |  |  |  |
| **Maternal education** | -0.0895 | 0.0325 | 0.669 | 1 |  |  |  |  |  |  |  |  |  |  |
| **P-value** | < 0.001 | 0.00367 | < 0.001 |  |  |  |  |  |  |  |  |  |  |  |
| **One-child family** | 0.0497 | -0.0877 | 0.198 | 0.201 | 1 |  |  |  |  |  |  |  |  |  |
| **P-value** | < 0.001 | < 0.001 | < 0.001 | < 0.001 |  |  |  |  |  |  |  |  |  |  |
| **Left-behind experience** | -0.0763 | -0.0187 | 0.0401 | 0.0211 | 0.0357 | 1 |  |  |  |  |  |  |  |  |
| **P-value** | < 0.001 | 0.0948 | < 0.001 | 0.0599 | 0.00145 |  |  |  |  |  |  |  |  |  |
| **Family structure** | -0.0336 | 0.0422 | 0.00242 | 0.0232 | -0.00182 | -0.212 | 1 |  |  |  |  |  |  |  |
| **P-value** | 0.00274 | < 0.001 | 0.829 | 0.038 | 0.871 | < 0.001 |  |  |  |  |  |  |  |  |
| **Family income** | -0.0511 | 0.00695 | 0.441 | 0.444 | 0.0611 | 0.0216 | -0.0515 | 1 |  |  |  |  |  |  |
| **P-value** | < 0.001 | 0.535 | < 0.001 | < 0.001 | < 0.001 | 0.0537 | < 0.001 |  |  |  |  |  |  |  |
| **Family function** | -0.0942 | -0.0395 | 0.065 | 0.0621 | 0.0688 | 0.179 | -0.0947 | 0.0545 | 1 |  |  |  |  |  |
| **P-value** | < 0.001 | < 0.001 | < 0.001 | < 0.001 | < 0.001 | < 0.001 | < 0.001 | < 0.001 |  |  |  |  |  |  |
| **Family history of mental illness** | 0.0224 | 0.00233 | -0.00356 | 0.0152 | -0.0326 | -0.0405 | 0.0303 | 0.00294 | -0.0784 | 1 |  |  |  |  |
| **P-value** | 0.0455 | 0.835 | 0.751 | 0.176 | 0.0036 | < 0.001 | 0.00684 | 0.793 | < 0.001 |  |  |  |  |  |
| **Adolescent mental health literacy** | 0.284 | 0.0717 | 0.0766 | 0.0693 | 0.0398 | -0.0316 | -0.0167 | 0.0514 | -0.0841 | -0.0164 | 1 |  |  |  |
| **P-value** | < 0.001 | < 0.001 | < 0.001 | < 0.001 | < 0.001 | 0.00476 | 0.135 | < 0.001 | < 0.001 | 0.144 |  |  |  |  |
| **Adolescent depression (PHQ-2)** | 0.0823 | 0.11 | 0.0365 | 0.0372 | -0.016 | -0.115 | 0.0693 | 0.0325 | -0.475 | 0.0779 | 0.11 | 1 |  |  |
| **P-value** | < 0.001 | < 0.001 | 0.00111 | < 0.001 | 0.153 | < 0.001 | < 0.001 | 0.00377 | < 0.001 | < 0.001 | < 0.001 |  |  |  |
| **Adolescent insomnia (ISI-7)** | 0.0223 | 0.0207 | 0.0393 | 0.0422 | -0.0276 | -0.108 | 0.0823 | 0.0287 | -0.4 | 0.0826 | 0.0514 | 0.515 | 1 |  |
| **P-value** | 0.0469 | 0.0642 | < 0.001 | < 0.001 | 0.0137 | < 0.001 | < 0.001 | 0.0103 | < 0.001 | < 0.001 | < 0.001 | < 0.001 |  |  |
| **Adolescent anxiety (GAD-7)** | 0.0752 | 0.131 | 0.0897 | 0.0901 | 0.00378 | -0.0938 | 0.0735 | 0.0716 | -0.443 | 0.0794 | 0.141 | 0.703 | 0.601 | 1 |
| **P-value** | < 0.001 | < 0.001 | < 0.001 | < 0.001 | 0.736 | < 0.001 | < 0.001 | < 0.001 | < 0.001 | < 0.001 | < 0.001 | < 0.001 | < 0.001 |  |

**Supplementary Table 2. Variance inflation factors (VIFs) for model 2**

| **Model2** |  | **VIF** |
| --- | --- | --- |
| High school (vs middle school) |  | 1.133 |
| Female (vs male) |  | 1.043 |
| Paternal education |  |  |
|  | High school diploma or below | - |
|  | Associate or bachelor's degree | 1.88 |
|  | Master's degree or above | 1.607 |
|  | Not sure | 6.01 |
| Maternal education |  |  |
|  | High school diploma or below | - |
|  | Associate or bachelor's degree | 1.889 |
|  | Master's degree or above | 1.477 |
|  | Not sure | 6.017 |
| One-child famiy (vs no) |  | 1.101 |
| Left-behind experience (vs no) |  | 1.125 |
| Family structure |  |  |
|  | Nuclear family | - |
|  | Stem family | 1.118 |
|  | Single-parent family | 1.13 |
|  | Reognization family | 1.02 |
|  | Grandparent family | 1.101 |
| Family income |  |  |
|  | ¥80,000-¥150,000/year | - |
|  | less than ¥80,000/year | 1.311 |
|  | ¥150,000-¥300,000/year | 1.378 |
|  | more than ¥300,000/year | 1.366 |
| Family function |  | 1.427 |
| Family history of mental illness (vs no) |  | 1.015 |
| Adolescent mental health litracy |  | 1.127 |
| Adolescent depression (PHQ-2) |  | 2.161 |
| Adolescent insominia (ISI-7) |  | 1.67 |
| Adolescent anxiety (GAD-7) |  | 2.443 |
